# Clinical and Molecular Epidemiology of Multidrug-Resistant *P*. *aeruginosa* Carrying *aac(6')-Ib-cr*, *qnrS1* and *bla*_SPM_ Genes in Brazil

**DOI:** 10.1371/journal.pone.0155914

**Published:** 2016-05-24

**Authors:** Bruna Fuga Araujo, Melina Lorraine Ferreira, Paola Amaral de Campos, Sabrina Royer, Deivid William da Fonseca Batistão, Raquel Cristina Cavalcanti Dantas, Iara Rossi Gonçalves, Ana Luiza Souza Faria, Cristiane Silveira de Brito, Jonny Yokosawa, Paulo Pinto Gontijo-Filho, Rosineide Marques Ribas

**Affiliations:** 1 Instituto de Ciências Biomédicas (ICBIM), Laboratory of Molecular Microbiology, Universidade Federal de Uberlandia (UFU), Uberlandia, Minas Gerais, Brazil; 2 Instituto de Ciências Biomédicas (ICBIM), Laboratory of Virology, Universidade Federal de Uberlandia (UFU), Uberlandia, Minas Gerais, Brazil; University of North Dakota, UNITED STATES

## Abstract

We described a comprehensive analysis of the molecular epidemiology of multidrug-resistant (MDR) *P*. *aeruginosa*. Molecular analysis included typing by Pulsed Field Gel Electrophoresis, identification of genes of interest through PCR-based assays and sequencing of target genes. Case-control study was conducted to better understand the prognostic of patients and the impact of inappropriate therapy in patients with bacteremia, as well as the risk factors of MDR infections. We observed a high rate of MDR isolates (40.7%), and 51.0% of them was independently associated with inappropriate antibiotic therapy. Bacteremia was detected in 66.9% of patients, and prolonged hospital stay was expressive in those resistant to fluoroquinolone. Plasmid-mediated quinolone resistance genes (PMQR), *qnrS*_*1*_ and *aac(6’)Ib-cr*, were detected in two different nosocomial isolates (5.3%), and the *aac(6’)-Ib*_*7*_ variant was detected at a high frequency (87.5%) in those negative to PMQR. The presence of mutations in *gyrA* and *parC* genes was observed in 100% and 85% of selected isolates, respectively. Isolates harboring PMQR genes or mutations in *gyrA* and *parC* were not closely related, except in those containing SPM (São Paulo metallo-β-lactamase) clone. In addition, there is no study published in Brazil to date reporting the presence of *Pseudomonas aeruginosa* isolates harboring both *qnrS*_*1*_ and *aac(6’)Ib-cr* genes, with alarming frequency of patients with inappropriate therapy.

## Introduction

Currently, the increasing incidence of MDR *P*. *aeruginosa* is a global problem as a consequence of the ability of this microorganism to develop resistance to almost all antibiotics that are available for treatment, either by mutations present in chromosomal gene, or by horizontal gene transfer [1,2]. In Brazil, this problem is aggravated because of either the very high density of the antimicrobials use, especially β-lactams (including carbapenems) and fluoroquinolones [3,4] and the lack of affirmative action’s for prevention of these infections [5,6].

Carbapenems are a therapeutic choice for the treatment of severe infections caused by *P*. *aeruginosa*. However, its use has been threatened mainly by the increased incidence of isolates resistant to this class of antibiotics [7–9]. Among the carbapenem resistance mechanisms, there is the presence of carbapenemases, which are a heterogeneous group of β-lactamases comprising Classes A (penicillinases), B (metalloenzymes) and D (oxacillinases), with the ability to hydrolyze imipenem and meropenem in addition to other penicillins and cephalosporins [10]. The genes encoding the carbapenemases are usually located in plasmids, which significantly increase the risk of their dissemination [11,12]. The carbapenem resistance in *P*. *aeruginosa* infection occurs mainly by metallo-β-lactamase (MBL), in addition to the appearance of other β-lactamases with carbapenemase activity, including KPC (*Klebsiella pneumoniae* carbapenemase) and OXA-carbapenemase [7,9,13,14].

In recent years, the production of MBL by *P*. *aeruginosa* isolates has assumed epidemiological importance and is associated with high mortality rates [15]. MBLs confer resistance to carbapenems, and are generally encoded by mobile elements facilitating the spread of antibiotic resistance [16–18]. The *bla*_SPM-1_ gene has been spread in Brazilian regions by the persistent MDR *P*. *aeruginosa*, and studies have reported concerns about its worldwide spread and potential pandemic [7, 18–20]. The increased prevalence of infections and colonization by *P*. *aeruginosa* São Paulo metallo-β-lactamases (SPM)-producers suggests that the gene *bla*_SPM-1_ settled successfully in plasmids and is associated with high risk of formation of clones, which can facilitate its global spread [21, 22].

Beyond the potent *in vitro* anti-pseudomonal activity, the use of fluoroquinolone antibiotics has spread widely in the past decade, and its usage popularity has also facilitated the emergence of resistant and MDR strains [23]. The resistance to fluoroquinolones is mainly due to (i) the point mutations in quinolone resistance determining regions (QRDR) in the DNA gyrase (*gyrA* and *gyrB*) and topoisomerase IV (*parC* and *parE*) genes, (ii) the presence of plasmid-mediated quinolone resistance (PMQR) determinants, and/or (iii) the decreased uptake of the drug due to the loss of a membrane-bound porin and/or drug extrusion via efflux pumps [24]. The co-existence of mutations in genes encoding for type-II topoisomerase and PMQR is often found together in microorganisms of the *Enterobacteriaceae* family, and the presence of PMQR determinants may promote QRDR mutations, increasing the resistance to fluoroquinolone [25, 26]. Although PMQR genes, such as *qnrA*, *qnrB*, *qnrC*, *qnrD*, *qnrS*, *qepA* and *aac(6’)-Ib-cr*, have been increasingly reported in bacterial pathogens within the *Enterobactericeae* family, they have not been detected frequently in *P*. *aeruginosa* isolates [24, 27]. To the best of our knowledge, as far as we know, this is the first description of the presence of PMQR genes in *P*. *aeruginosa* isolates in Brazil.

In addition, only a few studies have examined the consequences of resistance to antibiotics and the impact of inappropriate therapy on the outcome of patients with MDR *P*. *aeruginosa* infections. Thus, this study was performed to evaluate this factor, and to identify the risk factors in patients with MDR *P*. *aeruginosa* infections. In addition, we also aimed to determine: (i) the presence of metallo-β-lactamases genes in carbapenem-resistant isolates; (ii) the co-occurrence of PMQR determinants and altered *gyrA* and *parC* genes in fluoroquinolone-resistant isolates; and (iii) the pattern of clonal spread of *P*. *aeruginosa* in the hospital environment.

## Materials and Methods

### Study design and data collection

Active surveillance was conducted from May 2009 to December 2012 and from April to October 2014 for the detection of patients with *P*. *aeruginosa* infections resistant to carbapenems and fluoroquinolones at Uberlândia University Hospital (Brazil). In total, 242 episodes of *P*. *aeruginosa* infections obtained from 236 patients were included in the study. From this surveillance, two case-control studies were conducted: (i) to determine the risk factors associated with MDR *P*. *aeruginosa* infections, and (ii) to determine the risk factors associated with antimicrobial resistance and treatment outcome in patients with *P*. *aeruginosa* bacteremia. In both studies, only the first episode of each infection was considered. The demographic, clinical and epidemiological data of the patients were also obtained through review of medical records, following the model of National Healthcare Safety Network (NHSN).

### Definitions

According to the Centers for Disease Control and Prevention (CDC), bacteremia was defined as the presence of viable bacteria in the blood, documented by a positive blood culture result [28]. The isolates were considered to be nosocomial if the infection occurred >48 h after admission and no clinical evidence of infection on admission existed [29]. The criteria used for defining MDR phenotype was: non-susceptible to ≥1 agent in ≥3 antimicrobial categories [30]. Previous antibiotic use was considered when the patient received therapy with any antibiotic for at least 72 h over a period of 30 days prior to the microbiological infection diagnosis [31]. The antimicrobial therapy was considered to be appropriate if the initial antibiotics, which were administered within 24 h of acquisition of a blood culture sample, included at least one antibiotic that was active *in vitro* [32]. The 30-day mortality was considered as the number of deaths of patients with infections during hospitalization that occurred within 30 days of the diagnosis of infection [33], and the 5-day mortality, also known as early mortality, was considered as the number of deaths within 5 days of hospitalization [34]. Hospital stays were considered prolonged if they reached or exceeded 45 days [35]. It was considered MIC50 and MIC90 represent the concentration of antimicrobial agent (μg/mL) that inhibited 50% and 90%, respectively, of the isolates tested [36].

### Clinical microbiological and antibiotic resistant profile

Microbial identification and antimicrobial susceptibility tests were performed on a VITEK II system (bioMérieux, Brazil) for the following antimicrobials: aminoglycoside (gentamicin, amikacin), carbapenems (imipenem, meropenem), cephalosporin (cefepime), fluoroquinolone (ciprofloxacin) and penicillin plus β-lactamase inhibitors (piperacillin-tazobactam). Quality-control protocols were used according to the standards of the Clinical and Laboratory Standard Institute [37, 38]. The isolates with intermediate susceptibility were considered as resistant.

The minimum inhibitory concentration (MIC) and the confirmation test of resistance to imipenem (≥8 μg/mL) were performed by the E-test^®^ method, according to the manufacturer’s guidelines (AB Biodisk, Sweden) [38]. In addition, resistance to ciprofloxacin was confirmed by broth microdilution method according to Capuano [39] with modifications, and the interpretations also were made according to CLSI [38], considering resistance to ciprofloxacin ≥4 μg/mL.

### Characterization of strains harboring MBL and PMQR genes

Forty clinical *P*. *aeruginosa* fluoroquinolone-resistant isolates were selected, being obtained from 39 patients, with various clinical infections (urinary infection, pneumonia, wound infection, otitis, and bloodstream infection).

DNA extraction was performed using a PureYield^™^ Plasmid Miniprep System (Promega, Brazil). Amplification of the MBL and PMQR markers (*bla*_IMP_, *bla*_VIM_, *bla*_SPM,_ *bla*_GIM,_ *bla*_SIM,_ *qnrA*, *qnrB*, *qnrC*, *qnrD*, *qnrS*, *qepA* and *aac(6’)-Ib-cr*) were performed using primers listed in S1 Table (available in the Supporting Information). The reaction mixture (25 μL) contained 1.0 μL DNA template (10 ng), 12.5 μL GoTaq^®^ Green Master Mix (Promega) and 0.5 μL of each primer. Amplifications were performed in Mastercycler Personal (Eppendorf) using the following program: initial denaturation at 95°C for 2 min followed by 30 cycles of 30 seconds at 95°C, 1 min at annealing temperature (54°C for *bla*_IMP_, *bla*_VIM_, *bla*_SPM,_ *bla*_GIM_ and *bla*_SIM;_ 51°C for *qnrA*, *qnrB*, *qnrC*, *qnrS* and *qnrD*; 52°C for *qepA* and *aac(6’)-Ib-cr*), 1 min at 72°C and a final extension steap of 5 min at 72°C. Multiplex PCR was performed for genotypic characterization of different MBL (*bla*_IMP_, *bla*_VIM_, *bla*_SPM,_ *bla*_GIM_ and *bla*_SIM_) and PMQR (*qnrA*, *qnrB*, *qnrC*, and *qnrS*) genes; and, for the others genes individual PCRs were carried out (*qnrD*, *qepA* and *aac(6’)-Ib-cr*). The amplified PCR products were visualized by electrophoresis in 1.5% agarose gel by the photo documentation System L-Pix EX (Loccus Biotechnology, Brazil).

### Sequencing of PMQR genes and QRDRs mutations

The PCR products of target regions for QRDR mutations (*gyrA* and *parC*) and PMQR genes (*qnrS* and *aac(6’)-Ib*) were sequenced (primers listed in S1 Table), using an automatic sequencer ABI-PRISM 3100 Genetic Analyzer (Applied Biosystems, USA). Sequences were edited using SeqMan Pro alignment was carried out with MegaAlign, both of Lasergene package version 10 (DNAStar, USA) and deposited at GenBank (http://www.ncbi.nlm.nih.gov/genbank/) [accessions numbers: **KT962252** (*qnrS*_*1*_), **KT987419** (*aac(6’)-Ib-cr*) and **KT987420** (*aac(6’)-Ib*_*7*_)].

### Pulsed-Field Gel Electrophoresis (PFGE)

Isolates were typed according to the protocols described by Galetti [40] with modifications, following digestion of genomic DNA with *Spe*I restriction enzyme (Promega). DNA fragments were separated on 1% (w/v) agarose gels in 0.5x TBE [Tris–borate–ethylene diamine tetra-acetic acid (EDTA)] buffer using a CHEF DRIII apparatus (Bio-Rad, USA) with 6 V/cm, pulsed from 5 s to 40 s, for 21 h at 12°C. Gels were stained with ethidium bromide and photographed under ultraviolet light. Computer-assisted analysis was performed using BioNumerics 5.01 software (Applied Maths, Belgium). Comparison of the banding patterns was accomplished by the unweighted pair-group method with arithmetic averages (UPGMA) using the Dice similarity coefficient.

### Statistical analysis

The Chi-square or Fisher’s exact test was used to compare discrete variables. The comparison of two quantitative variables was made using the Mann–Whitney test for nonparametric variables and the Student t test for parametric variables. Two-sided tests were used for all analyses. Multivariate analysis was performed using multiple logistic regression and the values were included when significance was <0.05 in univariate analysis. To determine inappropriate therapy for mortality within 30 days of hospitalization, a multiple logistic regression model was used to control for the effects of confounding variables. All p-value <0.05 was considered statistically significant. The epidemiological data were analyzed through the programs Graph Pad Prism^®^ 5.0 (La Jolla, USA) and BioEstat 5.0 (Tefé, Brazil).

### Ethical considerations

The data and the samples analyzed in the present study were obtained in accordance with the norms and approved by the Federal University of Uberlandia Ethics Committee (UFU), through license number 36601814.7.0000.5152. For this study, samples were collected at the Microbiology Laboratory of the Clinical Hospital, with no contact to the patient and with the permission of the Hospital. Moreover, this study was retrospective and there are no patient identification when performed data collection, so the ethics committee dismissed the informed consent term and clarified.

## Results

**Fig 1:**
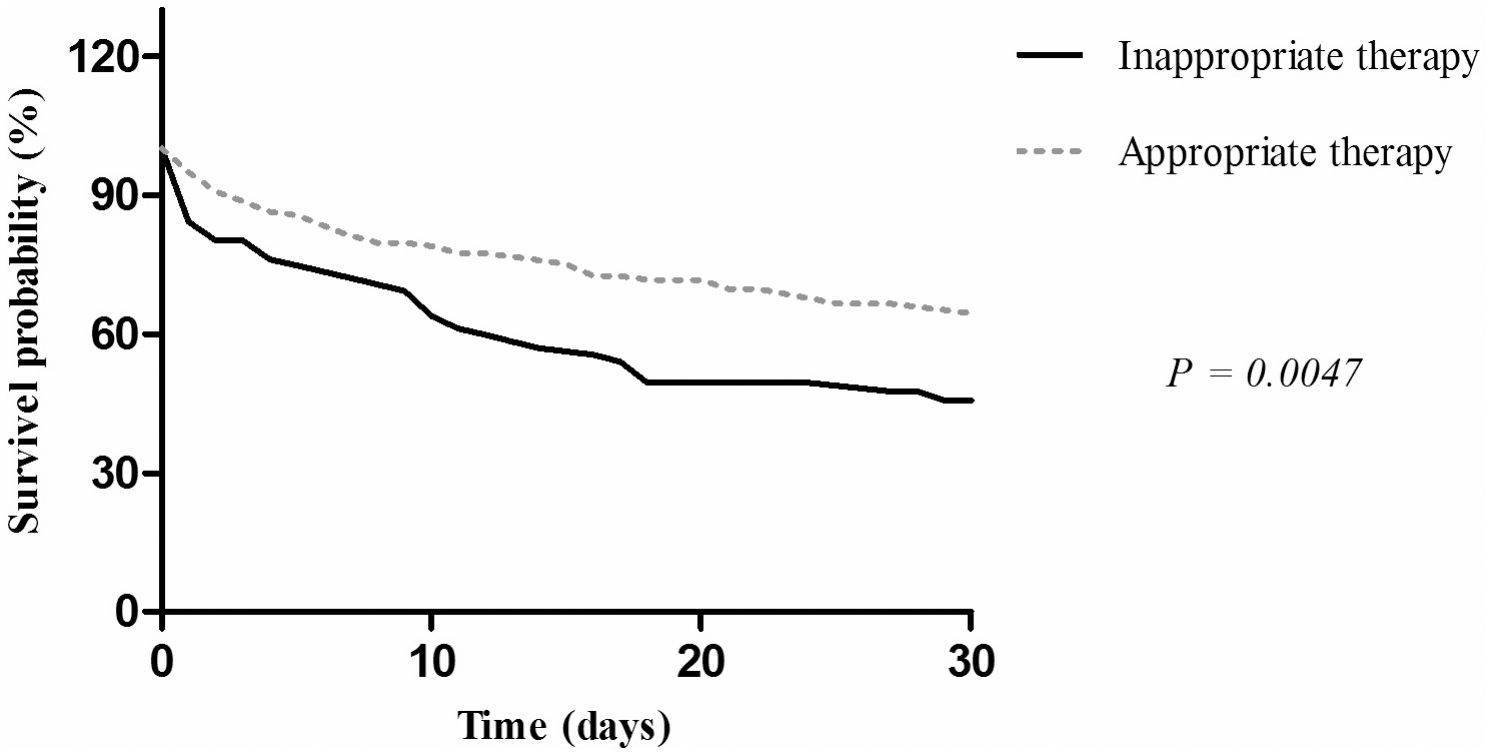
Survival curve using the Kaplan–Meier method for patients who received appropriate antimicrobial therapy compared with those who received inappropriate therapy. *P* ≤ 0.05 –statistically significant.

From May 2009 to December 2012 and from April to October 2014, a total of 236 non-repetitive patients with *P*. *aeruginosa* infections at the University Hospital were included in the study. The univariate analysis and independent risk factors associated with MDR *P*. *aeruginosa* infections are summarized in Table 1. According to antimicrobial susceptibility testing results, MDR *P*. *aeruginosa* infections occurred in 40.7% of the cases. Data from these patients (MDR) were compared with a sensitive *P*. *aeruginosa* infections group (non-MDR). In the whole series, prior exposure to carbapenems and inappropriate therapy as well as the co-morbidity condition (diabetes mellitus) were significant in the univariate analysis by MDR *P*. *aeruginosa* infections. The results of multivariate analyses showed that factors independently associated with MDR *P*. *aeruginosa* were patients who received inappropriate therapy. The Kaplan–Meier cumulative survival estimates (Fig 1) for patients with inappropriate versus appropriate therapy showed that the first group had a lower probability of survival than the group that received appropriate therapy (*P* = 0.0047). The 30-day mortality rate of the first group was 55.3%, whereas that of the second group was 34.4%. Furthermore, of the total mortality, 5-day mortality rate was 40.6% (54/133) independently of the therapy received.

**Table 1:** Univariate and multivariate analyses, mortality and independent risk factors associated with multidrug-resistant *P*. *aeruginosa* infections.

Risk factor/ characteristics	Total	MDR^1^	non-MDR	Univariate		Multivariate	
	n = 236 (%)	n = 96 (%)	n = 140 (%)	OR^2^ (CI^3^ 95%)	*P* value	OR (CI 95%)	*P* value
**Age [mean]; ±SD**^4^	52.7; ±22.9	56.5; ±18.3	50.6; ±25.1	-	0.2111	-	-
**Male**	166 (70.3)	65 (67.7)	101 (72.1)	0.81 (0.46–1.42)	0.4638	-	-
**Female**	70 (29.7)	31 (32.3)	39 (27.8)	1.23 (0.70–2.17)	0.4638	-	-
**Length of hospital stay days [mean]; ±SD**	55.0; ±69.7	54.7; ±46.5	53.3; ±80.9	-	0.0669	-	-
**Invasive procedures**	210 (88.9)	86 (89.6)	123 (87.9)	1.19 (0.52–2.72)	0.6824	-	-
Mechanical ventilation	126 (53.4)	58 (60.4)	68 (48.6)	1.62 (0.95–2.74)	0.0731	-	-
Tracheostomy	120 (50.8)	56 (58.3)	64 (45.7)	1.66 (0.98–2.81)	0.0568	-	-
Urinary catheter	163 (69.1)	71 (73.9)	92 (65.7)	1.48 (0.83–2.63)	0.1783	-	-
Central venous catheter	192 (81.4)	77 (80.2)	115 (82.1)	0.88 (0.45–1.71)	0.7078	-	-
Surgical drain	41 (17.4)	17 (17.7)	24 (17.1)	1.04 (0.52–2.06)	0.9103	-	-
Enteral probes/gastric nutrition	172 (72.9)	71 (73.9)	101 (72.1)	1.10 (0.61–1.97)	0.7580	-	-
Haemodialysis	71 (30.1)	29 (30.2)	42 (30.0)	1.01 (0.57–1.78)	0.9727	-	-
**Co-morbidity conditions**	146 (61.9)	57 (59.4)	89 (63.6)	0.84 (0.49–1.43)	0.5144	-	-
Heart failure	60 (25.4)	27 (28.1)	33 (23.6)	1.27 (0.70–2.29)	0.4300	-	-
Neoplasia	36 (15.2)	9 (9.4)	27 (19.3)	0.43 (0.19–0.97)	0.0375	-	-
Diabetes mellitus	40 (16.9)	22 (22.9)	18 (12.9)	2.01 (1.01–4.00)	0.0430*	1.9907 (0.97–4.09)	0.0608
Chronic renal failure	66 (28.0)	28 (29.2)	38 (27.1)	1.10 (0.62–1.97)	0.7337	-	-
**Previous use of antimicrobial**	195 (82.6)	82 (85.4)	113 (80.7)	1.40 (0.69–2.83)	0.3490	-	-
Carbapenems	119 (50.4)	56 (58.3)	63 (45.0)	1.71 (1.01–2.89)	0.0442*	0.8928 (0.51–1.55)	0.6873
Fluoroquinolone	51 (21.6)	24 (25.0)	27 (19.3)	1.41 (0.76–2.64)	0.2753	-	-
Cephalosporin (3 and 4th generation)	161 (68.2)	65 (67.7)	96 (68.6)	0.96 (0.55–1.68)	0.8888	-	-
**Inappropriate therapy**	85 (36.0)	49 (51.0)	36 (25.7)	3.01 (1.73–5.23)	< 0.0001*	3.0169 (1.72–5.31)	0.0001*
**Mortality**							
Total mortality	133 (56.4)	54 (56.2)	79 (56.4)	0.99 (0.59–1.68)	0.9783	-	-
30-day mortality	99 (41.9)	42 (43.7)	57 (40.7)	1.133 (0.67–1.92)	0.6425	-	-
5-day mortality	54 (22.9)	25 (26.0)	28 (20.0)	1.408 (0.76–2.61)	0.2746	-	-

^1^Multidrug-resistant;

^2^Odds ratio;

^3^Confidence interval;

^4^Standard deviation.

*P ≤ 0.05 –statistically significant for risk factor.

Antimicrobial therapy and clinical outcome of patients with or without bacteremia caused by *P*. *aeruginosa* were evaluated and it can be observed that patients with bacteremia caused by isolates resistant to carbapenems, had a high 5-day mortality rate. Moreover, the time of hospital stay was significantly higher for the MDR and fluoroquinolone-resistant groups when compared with susceptible group, and the latter was also independently associated in the multivariate analyses (Table 2).

**Table 2:** Antimicrobial therapy and clinical outcome of patients with bacteremia caused by *P*. *aeruginosa* resistant to carbapenems, fluoroquinolones and multiresistant.

	Patients with bacteremia	Patients without bacteremia	Univariate	
	n = 158 (%)	n = 78 (%)	*P value*	OR^1^ (CI^2^ 95%)
**Carbapenem resistant isolates**^3^				
Total	70 (100.0)	39 (100.0)	-	-
5-day mortality	22 (31.4)	5 (12.8)	0.0310*	3.117 (1.073–9.051)
30-day mortality	36 (51.4)	14 (35.9)	0.1188	1.891 (0.8453–4.229)
Inappropriate therapy	32 (45.7)	24 (61.5)	0.1131	0.5263 (0.2368–1.170)
Prolonged hospital stay^5^	40 (57.1)	15 (38.5)	0.0615	2.133 (0.9582–4.749)
**Fluoroquinolone resistant isolates**^4^				
Total	67 (100.0)	31 (100.0)	-	-
5-day mortality	21 (31.3)	4 (12.9)	0.0796	3.082 (0.9561–9.932)
30-day mortality	33 (49.2)	9 (29.0)	0.0600	2.373 (0.9534–5.904)
Inappropriate therapy	30 (44.8)	21 (67.7)	0.0343	0.3861 (0.1579–0.9440)
Prolonged hospital stay^6^	38 (56.7)	7 (22.6)	0.0016*	4.493 (1.701–11.86)
**Multidrug-resistant isolates**				
Total	67 (100.0)	29 (100.0)	-	-
5-day mortality	21 (31.3)	4 (13.8)	0.0820	2.853 (0.8809–9.241)
30-day mortality	32 (47.8)	11 (37.9)	0.3738	1.496 (0.6141–3.645)
Inappropriate therapy	30 (44.8)	19 (65.5)	0.0620	0.4267 (0.1727–1.055)
Prolonged hospital stay	39 (58.2)	7 (24.1)	0.0022*	4.378 (1.644–11.66)

^1^Odds ratio;

^2^Confidence interval;

^3^Imipenem and/or meropenem;

^4^Ciprofloxacin and/or norfloxacin;

^5^Length of stay ≥ 45 days;

^6^Significant risk factor by multivariate analyses *P =* 0.0088*, OR = 3.8151 (CI = 1.40–10.38); *P ≤ 0.05 –statistically significant.

The antimicrobial resistance analysis of the 242 *P*. *aeruginosa* isolates is presented in Table 3. The resistance to carbapenem and fluoroquinolones were the most frequents, 45.9% and 42.6% respectively, followed by resistance to aminoglycosides (38.4%), cephalosporin (3^rd^ and 4^th^ generation) (34.3%) and piperacilin/tazobactam (24.8%). No isolate was resistant to polymyxin.

**Table 3:** *Pseudomonas aeruginosa* antimicrobial resistance and sensitivity profiles to the main antimicrobials of isolates from 242 episodes of hospital infections.

Antimicrobials	Resistant		Sensitive	
	Isolates	Rate (%)	Isolates	Rate (%)
Cephalosporin (3^rd^ and 4^th^ generation)	83	34.3	159	65.7
Carbapenem	111	45.9	131	54.1
Aminoglycoside	93	38.4	149	61.6
Polymyxin	0	0	242	100.0
Piperacilin/Tazobactam	60	24.8	182	75.2
Fluoroquinolone	103	42.6	139	57.4

Table 4 summarizes the characterization of forty *P*. *aeruginosa* isolates that were resistant to fluoroquinolones (100%) and/or resistant to carbapenems (80%), with 87.5% (35/40) of nosocomial origin and 12.5% (5/40) classified as community acquired. The isolates were recovered from clinical specimens as blood (52.5%; 21/40), tracheal secretion (20.0%; 8/40), urine (15.0%; 6/40), tissue fragment (10.0%; 4/40) and otitis (2.5%; 1/40). All patients with bloodstream infection used a central venous catheter, and 50% of patients with urinary infection had a urinary catheter. Furthermore, 86.7% (13/15) of the patients with pneumonia had ventilator-associated pneumonia (VAP). Antimicrobial susceptibility results were analyzed and 87.5% (35/40) isolates was characterized as MDR. The resistance rates to carbapenem, cefepime, piperacillin/tazobactam, and aminoglycoside were 80% (32/40), 72.5% (29/40), 57.5% (23/40) and 55% (22/40), respectively (data not shown). Moreover, 40% community isolates were characterized as MDR. The minimum inhibitory concentrations of imipenem and ciprofloxacin to inhibit 90% of 38 *P*. *aeruginosa* isolates selected were ≥32 and 64 μg/mL, respectively.

**Table 4:** Characterization of *P*. *aeruginosa* isolates resistant to fluoroquinolones and/or carbapenems with regard to the minimum inhibitory concentration, *bla*_SPM_, *bla*_VIM_, *qnrS*, *aac(6´)-Ib-cr* and *aac(6´)-Ib*_*7*_ genes, quinolone resistance determining region mutations and the PFGE patterns.

Characteristics	Positive/analyzed (%)
Phenotype	
Multiresistant	35/40 (87.5)
Non-multiresistant	05/40 (12.5)
Metallo-β-lactamase	
SPM	05/32 (15.6)
VIM	02/32 (06.2)
PMQR/*aac*(6′)-*Ib*^1^	
*qnrS*_*1*_	*01/38 (02*.*6)*
*aac*(6′)-*Ib*-*cr*	*01/38 (02*.*6)*
*aac*(6′)-*Ib*_*7*_	*28/32 (02*.*6)*
QRDR^2^ mutations	
*gyrA*: Thr83Ile	20/20 (100.0)
*parC*: Ser87Leu	16/20 (80.0)
*parC*: Glu91Lys	01/20 (05.0)
*gyrA* + *parC*: Thr83Ile + Ser87Leu	16/20 (80.0)
*gyrA* + *parC*: Thr83Ile + Glu91Lys	01/20 (05.0)
PFGE^3^ patterns	
A	03/21 (14.3)
B	02/21 (09.5)
C	02/21 (09.5)
D-Q^4^	01/21 (04.8)
Predominant clones	
A, B and C	07/21 (33,3)
**Antimicrobial**	**MIC**^5^ **(μg/mL)**
Imipenem	
MIC_50_	≥32
MIC_90_	≥32
Ciprofloxacin	
MIC_50_	16
MIC_90_	64

^1^Plasmid-mediated quinolone resistance or aminoglycoside 6′-N-acetyltransferase type Ib;

^2^Quinolone-resistance determining region;

^3^Pulsed field gel electrophoresis;

^4^each clone;

^**5**^Minimum inhibitory concentration (n = 38). Of the total isolates, 12.5% are of Community origin (5/40).

Multiplex PCR to MBL was conducted only for carbapenem-resistant *P*. *aeruginosa* (32/40) from nosocomial and community infections, with 85.7% (30/35) and 40.0% (2/5), respectively, and seven isolates showed amplifications consistent with MBL genes indentified as *bla*_SPM-1_ and *bla*_VIM_ types.

The frequency of isolates that presented PMQR genes (*qnrS*_*1*_ and *aac(6’)-Ib-cr*) was 5.3% (2/38). The only MDR isolate of nosocomial origin that presented the *qnrS*_*1*_ gene was recovered from lung, with the following MIC for ciprofloxacin and imipenem, 64 μg/mL and ≥32 μg/mL, respectively. This isolate did not show to present the MBL genes tested (*bla*_IMP_, *bla*_VIM_, *bla*_SPM,_ *bla*_GIM_ and *bla*_SIM_). Overall, 85% (34/40) of the isolates harboured *aac(6’)-Ib* gene, and was recovered from blood (38.2%; 13/34), tracheal aspirate (32.4%; 11/34), urine (17.6%; 6/34) and tissue fragment (11.8%; 4/34). The sequencing results showed that only one nosocomial isolate, recovered from tissue fragment and showed non-MDR profile, presented the cr variant (*aac(6’)-Ib-cr* gene), with a MIC of 64 μg/mL for ciprofloxacin. Besides that, 87.5% (28/32) harboured the *aac(6’)-Ib*_*7*_ variant. None of the isolates harboured *qnrA*, *qnrB*, *qnrC*, *qnrD*, and *qepA* genes. Moreover, among PMQR-positive isolates none had community origin.

Of the 20 *P*. *aeruginosa* isolates evaluated for QRDR mutations in *gyrA* and *parC* (Thre83Ile; Ser87Leu; Glu91Lys), none presented concomitant PMQR determinants (Table 4).

**Fig 2:**
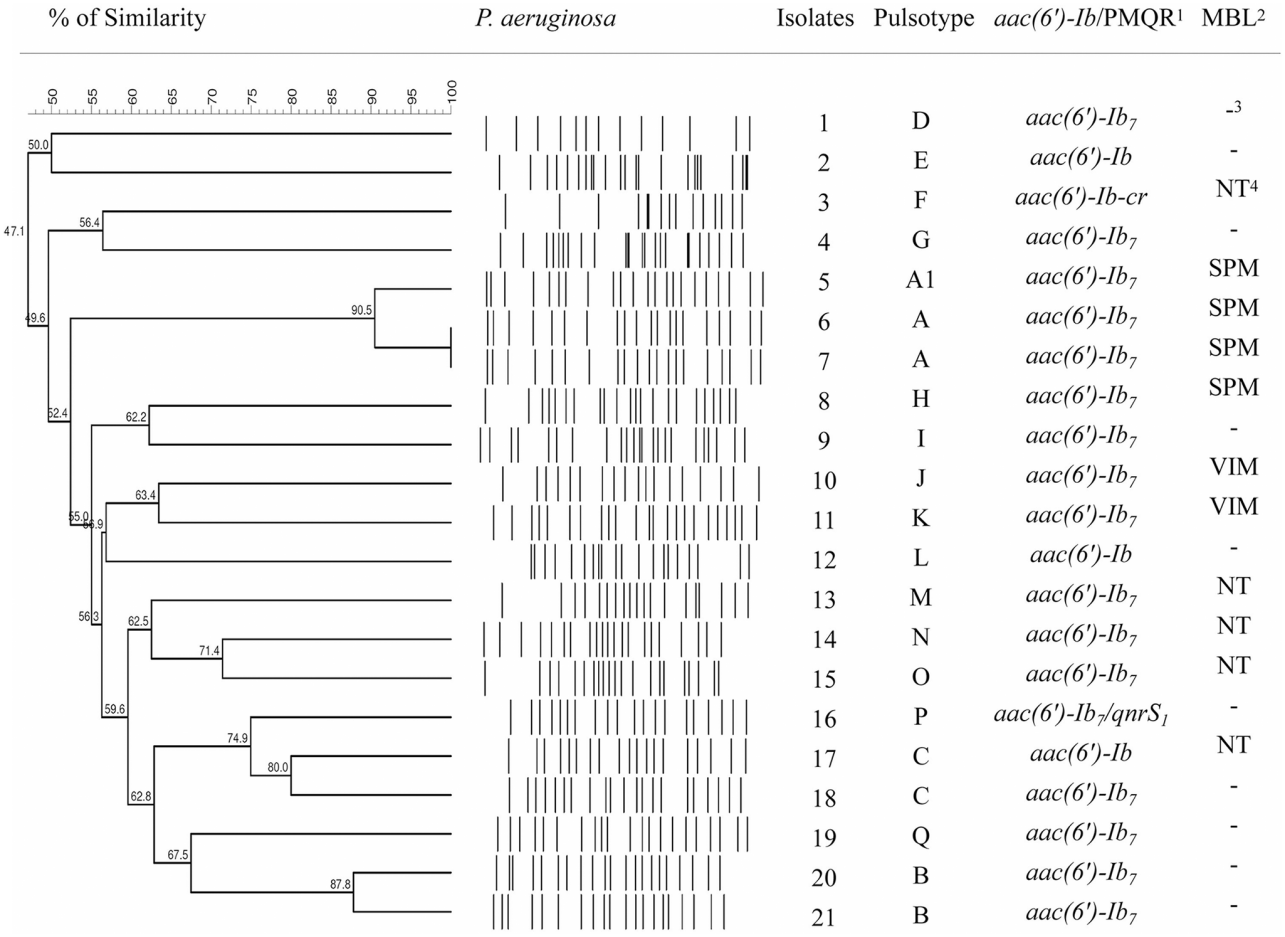
UPGMA dendrogram of PFGE profiles of 21 clinical *P*. *aeruginosa* isolates used in this study using the Dice coefficient under 1% tolerance and 1% optimization. A similarity coefficient of 80% was chosen for cluster definition. ^1^Plasmid-mediated quinolone resistance determinants or aminoglycoside 6′-N-acetyltransferase type Ib; ^2^Metallo-β-lactamase; ^3^negative; ^4^not tested.

A total of 17 PFGE patterns (A-Q) of *P*. *aeruginosa* were observed among the 21 isolates analyzed, comprising 3 main clones: A (14.3%; 3/21), B (9.5%; 2/21), C (9.5%; 2/21) (Table 4 and Fig 2). The pulsotype A had two subtypes (A and A1), and they belonged to isolates that contained the *bla*_SPM_ gene, while B and C had only one.

## Discussion

Recent studies have shown that multi-drug resistance and several virulence determinants are key factors that contribute to the global spread of *P*. *aeruginosa* in hospitals [41–43]. The present study evaluated the risk factors for the development of infections caused by MDR *P*. *aeruginosa*, as well as those associated with antimicrobial resistance and treatment outcome in patients with bacteremia. The independent risk factors for development of MDR *P*. *aeruginosa* infections include prior use of antibiotics (carbapenems, fluoroquinolones, broad-spectrum cephalosporins, and aminoglycosides), being bedridden or in the intensive care unit, prolonged hospital stay, *P*. *aeruginosa* infection or colonization within a previous period of one year, malignant disease, mechanical ventilation, and history of chronic obstructive pulmonary disease [44, 45]. Data of univariate analysis from this study have corroborated some of these risk factors. However, only inappropriate therapy was a risk predictor independent associated for developing MDR *P*. *aeruginosa* infections, but it must be considered as such within the epidemiological context [46]. Some studies provide evidences of a general view that the development of MDR can be caused by treatment that is inappropriate, incorrect and widespread use of carbapenems [3, 46]. The pressure of carbapenems use has contributed to an explosive increase of KPC (carbapenemase-producing *K*. *pneumoniae*), which has been responsible for 70.9% of *K*. *pneumoniae* infections in the hospital of this study (data not shown).

Among patients with bacteremia (158 patients), 44.3% had isolates resistant to carbapenems, 42.4% resistant to fluoroquinolones and 42.4% with a multidrug-resistant profile. The association of bacteremia with antimicrobial resistant isolates is common, but few studies have addressed this problem systematically [47–49]. In hospitalized patients, the association between bacteremia and fluoroquinolone-resistant *P*. *aeruginosa* was observed in our study with a high frequency of patients remained hospitalized longer (56.7%), independently associated with bacteremia. Similar frequencies were also observed for those patients with MDR isolates (58.2%). In the carbapenem-resistant group, was observed a higher early mortality rate (5 days) that was statistically significant with those that had bacteremia infections. These aspects have often been observed in developing countries like Brazil [48, 50, 51], where the macro and micro-regional differences in relation to the hospitals are extremely significant, as well as the characteristics of the hospitalized population [29, 52]. Besides, the lack of the microbiology laboratories and human and financial resources, a result of poor implementation of control practices for prevention of nosocomial infections, favors the intra and inter-hospital transmission of resistant pathogens that exhibit adaptation to the environment [6, 52]. Another aspect that must be considered is that the antimicrobial use is commonly abusive, empirical and often less judicious [6, 52]. A study performed by Dantas [53] in the same university hospital of this study, showed that the density of antibiotic use was much higher when compared with hospitals with similar size in other countries, allowing MDR strains such as Gram-negative non-fermentative bacteria to emerge and spread quickly [54].

In addition to the multi-drug resistance, special attention was given in this study to *P*. *aeruginosa* resistant to carbapenems, considering the significant increase of this resistance in Latin America and widespread of different clones associated with the production enzymes of the MBL type [55–58]. The predominant MBL-encoding gene in Brazil is *bla*_SPM-1_, which has been disseminated by the MDR *P*. *aeruginosa* clone SP/ST277, considered a high-risk clone [7, 18, 59, 60]. The *bla*_SPM-1_ gene was first detected in São Paulo, and later in others cities in Brazil, moreover several studies have shown its global spread and pandemic potential, causing important morbidity and mortality in hospital infections [18, 43, 60, 61]. In our study, we observed a high frequency of SPM among the isolates, especially those belonging to clone A. In addition, two isolates harboring *bla*_VIM_ were detected. An increase in the rates of *P*. *aeruginosa* isolates containing the gene *bla*_VIM_ has been also observed in others hospitals in Brazil [62, 63]. The frequent data among different types of metallo-β-lactamase in Brazil, and not in other countries, especially those developed, suggest the spread of specific clones and the knowledge of these facts may contribute to improving the multidrug resistance scenario.

Besides the carbapenem resistance, resistance to fluoroquinolones has become an increasing problem, so far, only a few studies have investigated the occurrence of PMQR in *P*. *aeruginosa* [27, 29, 64, 65]. PMQR is an important phenomenon that is being disseminated worldwide and the most relevant PMQR genes to date are the *aac(6’)-Ib-cr*, *qnr* and genes encoding efflux pumps such as *qepA* [24]. Surprisingly, the results from this study demonstrated for the first time the presence of PMQR genes in clinical isolates of *P*. *aeruginosa* in Brazil (5.3%), as well as a very significant high frequency not shown in other studies of the *aac(6’)-Ib*_*7*_ variant. The PMQR rate in our study was higher than that some reported in the literature [27, 65]. According to the study reported by Jiang et al. [27], the frequency of clinical isolates carrying the *aac(6’)-Ib-cr* gene was 1.9% (2/106 isolates), and the total rate of PMQR determinants was 3,8% among *P*. *aeruginosa* isolates, while in the Yang and colleagues [65] study, only one in 256 *P*. *aeruginosa* isolates (0.4%) showed a PMQR gene. The higher detection frequency of these genes in our study reflects most likely the complexity of epidemiology and resistance mechanisms associated with *P*. *aeruginosa* in developing countries. The co-existence of different PMQR genes in the same clinical isolate was not observed throughout this study, although it has been reported by Jiang et al. [27].

Regarding the target site mutations in QRDR of fluoroquinolone resistant *P*. *aeruginosa*, we observed mutations consistent with those published previously in all isolates tested [66–68]. Of total, 20 *Pseudomonas aeruginosa* of nosocomial origin were evaluated for mutations in *gyrA* and *parC*, and none of them presented PMQR genes. However, according to literature evidences, the chromosomal QRDR mutations in *gyrA* and *parC* genes are crucial to fluoroquinolone resistance, and their association with PMQR determinants may have an additional role that contributes to resistance to fluoroquinolones [26].

Another interesting observation from this study was the identification of a mixed wound infection by *P*. *aeruginosa* and *E*. *coli* in the patient who presented *P*. *aeruginosa* harboring the PMQR gene *aac(6’)-Ib-cr*. In a previous evaluation of *E*. *coli* infections in the same hospital, 71.4% had *aac(6’)-Ib-cr* gene (data not shown). The epidemiology of *P*. *aeruginosa* in this hospital proved to be complex and can be explained by its involvement in the propagation through transference of plasmids to different species as well as by clonal spread.

PFGE results, in this study, suggested that *P*. *aeruginosa* isolates harboring PMQR genes or mutations in *gyrA* and *parC* were not closely related, except in those containing SPM clone, wherein in the literature showing results with a similarity clonal among isolates of MDR *P*. *aeruginosa* [69].

In conclusion, our results confirm previous findings regarding the dissemination of SPM-type clones in Brazil, and contribute whit additional evidence that may indicate that inappropriate therapy may be a crucial factor to the emergence of MDR isolates, besides being related to worse prognosis. Additionally, this study demonstrates for the first time in Brazil the presence of the PMQR determinants in *P*. *aeruginosa*, spreading in the hospital. Future follow-up surveillance studies of molecular epidemiology in Brazilian hospitals have crucial importance to infection-control practices and reduce the effects of these infections on hospital patients.

## Supporting Information

S1 Table Primer nucleotide sequences and amplicon sizes of the PCR carried out for the detection and/or sequencing of antimicrobial resistance genes in this study. (DOC)
