# Supplementary material for: Clinical and Molecular Epidemiology of Multidrug-Resistant P. aeruginosa Carrying aac(6')-Ib-cr, qnrS1 and blaSPM Genes in Brazil
Source: PLoS One. 2016 May 24;11(5):e0155914. doi: 10.1371/journal.pone.0155914 (PMC4878783; doi:10.1371/journal.pone.0155914)
Supplement: S1 Table — (DOC) [file pone.0155914.s001.doc]

S1 Table. Primer nucleotide sequences and amplicon sizes of the PCR carried out for the detection and/or sequencing of antimicrobial resistance genes in this study

| **Genotype** | **Target gene** | **Sequence (5’– 3’)** | **Product Size (bp)** | **Reference** |
| --- | --- | --- | --- | --- |
| PMQR^1^ | *aac(6’)-Ib*  *aac(6’)-Ib-cr*^4^ | TTGCGATGCTCTATGAGTGGCTA  CTCGAATGCCTGGCGTGTTT CGTCACTCCATACATTGCAA | 482  - | [70] |
|  | *qnrA* | ATTTCTCACGCCAGGATTTG  GATCGGCAAAGGTTAGGTCA | 516 | [71] |
|  | *qnrB* | GATCGTGAAAGCCAGAAAGG  ATGAGCAACGATGCCTGGTA | 476 | [71] |
|  | *qnrC* | GGGTTGTACATTTATTGAATCG  CACCTACCCATTTATTTTCA | 307 | [71] |
|  | *qnrD* | CGAGATCAATTTACGGGGAATA  AACAAGCTGAAGCGCCTG | 565 | [72] |
|  | *qnrS* | GCAAGTTCATTGAACAGGGT  TCTAAACCGTCGAGTTCGGCG | 428 | [71] |
|  | *qepA* | AACTGCTTGAGCCCGTAGAT  GTCTACGCCATGGACCTCAC | 596 | [71] |
| MBL^2^ | *bla*_SPM_ | CTAAATCGAGAGCCCTGCTTG  CCTTTTCCGCGACCTTGATC | 798 | [21] |
|  | *bla*_IMP_ | GAATAGRRTGGCTTAAYTCTC  CCAAACYACTASGTTATC | 188 |  |
|  | *bla*_VIM_ | GTTTGGTCGCATATCGCAAC  AATGCGCAGCACCAGGATAG | 382 |  |
|  | *bla*_GIM_ | TCAATTAGCTCTTGGGCTGAC  CGGAACGACCATTTGAATGG | 72 |  |
|  | *bla*_SIM_ | GTACAAGGGATTCGGCATCG  TGGCCTGTTCCCATGTGAG | 569 |  |
| QRDR^3*^ | *gyrA* | GACGGCCTGAAGCCGGTGCAC  GCCCACGGCGATACCGCTGGA | 417 | [68] |
|  | *parC* | CGAGCAGGCCTATCTGAACTAT  GAAGGACTTGGGATCGTCCGGA | 186 |  |

^1^Plasmid mediated quinolone resistance. ^2^Metallo-β-lactamases. ^3^Quinolone resistance-determining regions. ^4^Sequencing. *Described and analyzed by Ferreira et al.[51]

References

21. Woodford N. Rapid Characterization of β-lactamases by Multiplex PCR. In: Gillespie SH, Mchugh TD. Antibiotic Resistance Protocols: Second Edition, Methods in Molecular Biology. 2010; 642: 181-192.

68. Lee JK, Lee YS, [Park YK](http://www.ncbi.nlm.nih.gov/pubmed?term=Park YK%5BAuthor%5D&cauthor=true&cauthor_uid=15784307), [Kim BS](http://www.ncbi.nlm.nih.gov/pubmed?term=Kim BS%5BAuthor%5D&cauthor=true&cauthor_uid=15784307). Alterations in the GyrA and GyrB subunits of topoisomerase II and the ParC and ParE subunits of topoisomerase IV in ciprofloxacin-resistant clinical isolates of *Pseudomonas aeruginosa*. [Int J Antimicrob Agents](http://www.ncbi.nlm.nih.gov/pubmed/12727072) 2005; 25: 290-295.

70. Park CH, Robicsek A, Jacoby JA, Sahm D, Hooper DC. Prevalence in the United States of *aac(6)-Ib-cr* Encoding a Ciprofloxacin-Modifying Enzyme. Antimicrobial Agents and Chemotherapy 2006; 50: 3953–3955.

71. Kim HB, Park CH, Kim CJ, Kim E, Jacoby GA, Hooper DC. Prevalence of Plasmid-Mediated Quinolone Resistance Determinants over a 9-Year Period. Antimicrob Agents Chemother. 2009; 53: 639–645.

72. Hu Y, Cai J, Zhang R, Zhou H, Sun Q, Chen G. Emergence of *Proteus mirabilis* Harboring blaKPC-2 and qnrD in a Chinese Hospital. Antimicrob Agents Chemother. 2012; 56: 2278–2282.
